# Cas historique de carcinome basocellulaire du nez

**DOI:** 10.11604/pamj.2014.19.326.4393

**Published:** 2014-11-27

**Authors:** Inssaf Ramli, Badredine Hassam

**Affiliations:** 1 Service de Dermatologie et Vénérologie, CHU Ibn Sina, Université Mohammed V, Souissi, Rabat, Maroc

**Keywords:** Carcinome basocellulaire infiltrant, Tumeur historique, pointe du nez, Carcinome basocellulaire infiltrant, Tumeur historique, pointe du nez

## Image en medicine

Le carcinome basocellulaire infiltrant est un carcinome mutilant de mauvais pronostic qui représente 40% des carcinomes de la pointe et l'aile du nez. Il s'agit de carcinome ulcéreux creusant de grands cratères nécrotiques qui finissent par envahir et effondrer les cavités nasosinusiennes et orbitaires ou perforer les os du crâne jusqu'aux méninges, ou détruire les vaisseaux du cou. Histologiquement, il est caractérisépar un aspect agressif avec une structure trabéculaire infiltrant faite des cellules souvent indifférenciées. La localisation au niveau du nez représente un réel challenge pour la prise en charge thérapeutique. Nous rapportons le cas d'une patiente de 67 ans, sans antécédents notables. Elle consultait pour une tumeur ulcéro-bourgeonnante croûteuse de contours perlés, au dépend de la pyramide nasale mettant à nu la muqueuse nasale. Cette tumeur évoluant depuis 10 ans était douloureuse et d'odeur nauséabonde. Les aires ganglionnaires étaient libres. L'histologie de la bordure de l'ulcération était en faveur d'un carcinome basocellulaire infiltrant. Une tomodensitométrie du massif facial a révélé un processus tumoral infiltrant avec envahissement du tissu sous-cutané adjacent et lyse osseuse (os propre du nez et maxillaire supérieur). Une chirurgie de réparation par lambeau de rotation frontonasal suivie par une radiothérapie étaient recommandées.

**Figure 1:**
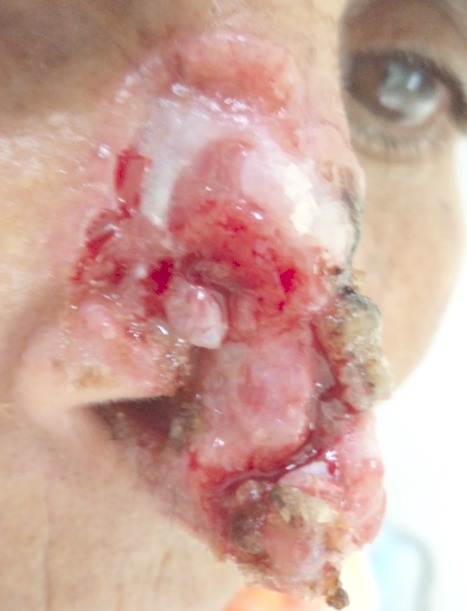
Tumeur mutilante du nez

